# Supplementary material for: A UMLS-based spell checker for natural language processing in vaccine safety
Source: BMC Med Inform Decis Mak. 2007 Feb 12;7:3. doi: 10.1186/1472-6947-7-3 (PMC1805499; doi:10.1186/1472-6947-7-3)
Supplement: Additional file 13 — RAP application directory. Sets up directory for RAP (RDF application for PHP) source code [file 1472-6947-7-3-S13.gz › rap/doc/phpdoc/blank.html]

RAP - Rdf API for PHP V0.9


# RAP - Rdf API for PHP

RAP is a software package for parsing, searching, manipulating, serializing and serving RDF models.

Its features include:

- statement-centric methods for manipulating an RDF model as a set of RDF triples- resource-centric methods for manipulating an RDF model as a set of resources- ontology-centric methods for manipulating an RDF model through vocabulary specific methods- quad- and named graph-centric Dataset API for manipulating sets of named RDF models- integrated RDF/XML, N3 and N-TRIPLE parser- integrated RDF/XML, N3 and N-TRIPLE serializer- in-memory or database model storage- support for the RDQL query language- inference engine supporting RDF-Schema reasoning and some OWL entailments
                - integrated RDF server providing similar functionality as the Joseki RDF server
                - graphical user-interface for managing database-backed RDF models
                - support for common vocabularies

Tutorials can be found here.

RAP consists of the following packages:

- Model
- RDF Dataset
- InfModel
- OntModel
- ResModel
- RDQL
- Syntax
- Utility
